# Supplementary material for: Resolving the full spectrum of human genome variation using Linked-Reads
Source: Genome Res. 2019 Apr;29(4):635–45. doi: 10.1101/gr.234443.118 (PMC6442396; doi:10.1101/gr.234443.118)
Supplement: Supplemental Material [file supp_gr.234443.118_Supplemental_longranger-2.2.zip › longranger-2.2.tar.bz2!/lr2.2-public/lib/stan/cmdstan/stan/doc/api/html/agrad_2rev_2error__handling_2matrix_2check__pos__definite_8hpp_source.html]

Stan: src/stan/agrad/rev/error\_handling/matrix/check\_pos\_definite.hpp Source File


|  |  |
| --- | --- |
|  | Stan  2.5.0  probability, sampling & optimization |


- Main Page
- Related Pages
- Namespaces
- Classes
- Files

- File List
- File Members

All Classes Namespaces Files Functions Variables Typedefs Enumerations Enumerator Friends Macros Pages

- src
- stan
- agrad
- rev
- error\_handling
- matrix

check\_pos\_definite.hpp

Go to the documentation of this file.

1 #ifndef STAN\_\_AGRAD\_\_REV\_\_ERROR\_HANDLING\_\_MATRIX\_\_CHECK\_POS\_DEFINITE\_HPP

2 #define STAN\_\_AGRAD\_\_REV\_\_ERROR\_HANDLING\_\_MATRIX\_\_CHECK\_POS\_DEFINITE\_HPP

3

4 #include <stan/agrad/rev/numeric\_limits.hpp>

5 #include <stan/agrad/rev/var.hpp>

6 #include <stan/math/error\_handling/matrix/check\_pos\_definite.hpp>

7 #include <stan/math/matrix/Eigen.hpp>

8 #include <stan/math/matrix/meta/index\_type.hpp>

9

10 namespace stan {

11

12  namespace agrad {

13

14  template <typename T\_result, class Policy>

15  inline bool check\_pos\_definite(const char\* function,

16  const Eigen::Matrix<var,Eigen::Dynamic,Eigen::Dynamic>& y,

17  const char\* name,

18  T\_result\* result,

19  const Policy&) {

20  using Eigen::Matrix;

21  using Eigen::Dynamic;

22  using stan::math::index\_type;

23  typedef typename index\_type<Matrix<double,Dynamic,Dynamic> >::type size\_type;

24  Matrix<double,Dynamic,Dynamic> y\_d(y.rows(),y.cols());

25  for (size\_type i = 0; i < y\_d.rows(); i++)

26  for (size\_type j = 0; j < y\_d.cols(); j++)

27  y\_d(i,j) = y(i,j).val();

28  return stan::math::check\_pos\_definite(function,y\_d,name,result,Policy());

29  }

30

31  }

32

33 }

34

35 #endif

check\_pos\_definite.hpp

stan::math::index\_type

Primary template class for the metaprogram to compute the index type of a container.

**Definition:** index\_type.hpp:21

stan::agrad::size\_type

Eigen::Matrix< double, Eigen::Dynamic, Eigen::Dynamic >::Index size\_type

**Definition:** typedefs.hpp:14

stan::math::check\_pos\_definite

bool check\_pos\_definite(const char \*function, const Eigen::Matrix< T\_y, Eigen::Dynamic, Eigen::Dynamic > &y, const char \*name, T\_result \*result)

Return true if the specified matrix is positive definite.

**Definition:** check\_pos\_definite.hpp:29

index\_type.hpp

var.hpp

Eigen.hpp

numeric\_limits.hpp

stan::agrad::check\_pos\_definite

bool check\_pos\_definite(const char \*function, const Eigen::Matrix< var, Eigen::Dynamic, Eigen::Dynamic > &y, const char \*name, T\_result \*result, const Policy &)

**Definition:** check\_pos\_definite.hpp:15


---

[ Stan Home Page ]

*© 2011–2014,
Stan Development Team.*
